# The role of the pulmonary function laboratory in the management of non-cystic fibrosis bronchiectasis

**DOI:** 10.36416/1806-3756/e20250529

**Published:** 2026-03-05

**Authors:** Rodrigo Abensur Athanazio

**Affiliations:** 1. Unidade de Doencas de Vias Aereas, Divisao de Pneumologia, Instituto do Coracao - InCor - Hospital das Clinicas, Faculdade de Medicina, Universidade de Sao Paulo - HCFMUSP - Sao Paulo (SP) Brasil.

## BACKGROUND

Bronchiectasis is a chronic pulmonary condition defined by a persistent and abnormal dilatation of the bronchial lumen. It is a heterogeneous condition and is typically characterized by frequent cough with productive sputum and recurrent exacerbations. Pulmonary function tests play an essential role in evaluating disease severity, assessing future risk, and guiding pharmacological treatment.^1^

## OVERVIEW

A 48-year-old never-smoking woman reported a 20-year history of chronic cough, which was frequently associated with sputum production and recurrent exacerbations requiring different antibiotics. Over the years she complained about progressive shortness of breath. She had no history of childhood asthma. In the previous year she had experienced an episode of hemoptysis leading to hospitalization. A chest CT scan revealed diffuse cylindrical bronchiectasis in all lung lobes, with cystic dilatations mainly in the lower lobes. Spirometry showed moderate (and similar) decreases in FEV_1_ and FVC, with normal FEV_1_/FVC ratio. After bronchodilator administration, there was no increase in FEV_1_ or FVC. Lung volume assessment by plethysmography identified air trapping caused by a high RV/TLC ratio, with preserved TLC. DL_CO_ was slightly reduced. 

Nonspecific ventilatory defect is common in patients with bronchiectasis, and a more in-depth assessment of pulmonary function is essential for improved disease characterization. 

Although bronchiectasis is defined by irreversible dilatation of the airways, it is crucial to consider the degree of small airway involvement in such patients. Despite dilatation of the more proximal airways, patients with bronchiectasis often exhibit significant obstructive impairment of the small airways, either due to mucoid impaction or chronic inflammation leading to constrictive bronchiolitis.^2^ Nevertheless, disease progression in patients with bronchiectasis may also result in a restrictive pattern with airflow limitation. As bronchiectatic areas progress, cystic formations may replace regions of healthy lung parenchyma, or areas of fibroatelectasis may develop, both contributing to a reduction in ventilated lung areas (Chart 1).^3^

**Chart 1:** Recommendations and key messages for lung function assessment based on the Brazilian consensus on non-cystic fibrosis bronchiectasis.^1^

Main recommendation	Perform spirometry with bronchodilator use every 6 months, lung volume assessment (if available) every 12 months, and the six-minute walk test at the physician’s discretion.
Spirometry	All bronchiectasis patients should undergo periodic functional assessment to detect any sign of decreased pulmonary function as early as possible. Spirometry with bronchodilator use is satisfactory in the vast majority of cases. Obstructive lung disease is the most common finding, but significant reductions in FVC can be found in more advanced disease, with increased lung parenchymal destruction. Decreased FEV_1_ correlates with the presence of dyspnea-as assessed by the mMRC scale-and the extent of disease on HRCT.
Complete PFT	The RV/TLC ratio is increased (suggesting air trapping), and TLC is normal or low. DL_CO_ test results are usually normal; DL_CO_ may be reduced in advanced disease and in the presence of associated emphysema.
Field tests	The six-minute walk test and the incremental shuttle walk test can provide additional functional information to spirometry. The six-minute walk distance correlates better with quality of life than with functional tests. The shuttle test can be useful especially in patients with preserved pulmonary function, because of the potential “ceiling effect” of the six-minute walk test, and has been validated for use in bronchiectasis patients.

mMRC: modified Medical Research Council; and PFT: pulmonary function testing.

In the context of this complex functional scenario, the use of field tests and exercise assessment may provide clinically relevant prognostic information (Chart 1). Field and exercise tests allow a more integrated patient assessment by incorporating multisystem involvement and associated comorbidities, which are frequently present in this population.^1^

Pulmonary function is an independent predictor of mortality and exacerbation risk in patients with bronchiectasis. The two most widely used multidimensional prognostic scores in bronchiectasis (the Bronchiectasis Severity Index and the Exacerbation in the previous year, FEV_1_, Age, Colonization, Extension, and Dyspnea score) incorporate spirometric parameters as key components of their assessment. Individuals with reduced pulmonary function-particularly those with an FEV_1_ of < 50% of the predicted value-have a higher five-year mortality risk.^4^ ^,^ ^5^ In addition, reduced pulmonary function is associated with poorer symptom control and, consequently, worse quality of life.^3^

## CLINICAL MESSAGE

Clinicians should adopt a comprehensive and integrated approach when interpreting functional assessment in patients with bronchiectasis. Although obstructive ventilatory impairment is the most common functional abnormality in such patients, mixed ventilatory defects are frequently observed, reflecting the presence of concomitant restrictive areas.^3^ Beyond spirometry, lung volume assessment, DL_CO_ measurement, and exercise testing may provide additional valuable information and complement functional evaluation. 

In patients with bronchiectasis, pulmonary function testing contributes not only to the initial assessment of disease severity but also to long-term follow-up, allowing monitoring of clinical and functional stability as well as evaluation of response to therapeutic interventions. Finally, pulmonary function parameters are closely associated with prognosis and play a key role in identifying patients at higher risk, supporting the selection of individuals who may benefit from a more aggressive therapeutic approach within the available treatments. 
